# Manipulating Electronic Effect of Nitrogen Donor-Based Ligands for Efficient Complexation and Separation of Palladium from Highly Acidic Solution

**DOI:** 10.3390/molecules30071533

**Published:** 2025-03-30

**Authors:** Yuyang Gan, Yimin Cai, Song Huang, Xiaowei Li, Wen Feng, Lihua Yuan

**Affiliations:** College of Chemistry, Key Laboratory of Radiation Physics and Technology of the Ministry of Education, Institute of Nuclear Science and Technology, Sichuan University, Chengdu 610064, China; 19981458619@163.com (Y.G.); ymcai@scu.edu.cn (Y.C.); anonunknown@163.com (S.H.); wfeng9510@scu.edu.cn (W.F.)

**Keywords:** nitrogen-based ligand, Pd(II) complexation, electronic effect, solvent extraction, high-level liquid waste

## Abstract

Nitrogen donor-based ligands are highly promising extractants for palladium separation from high-level liquid waste (HLLW). However, the electronic effect of these ligands, a critical factor influencing their complexation ability with Pd(II), remains largely unexplored. Herein, three picolinamide-based ligands were designed and synthesized, each featuring substituents with distinct electronic effects at the *para*-position of the pyridine (electro-donating methoxyl group for L-I, hydrogen for L-II, and electro-withdrawing ester group for L-III). The concurrent processes of Pd(II) coordination and ligand protonation enable the manipulation of pyridine nitrogen electronegativity, resulting in a tunable Pd(II) extraction performance. Notably, L-I exhibits the highest extraction efficiency at low acidities (≤1 M HNO_3_) but the lowest extraction at high acidities (≥3 M HNO_3_), whereas L-III shows the poorest efficiency at low acidities but the best performance at high acidities. The Job plot analysis and ESI-HRMS results reveal a 1:1 and 2:1 (L/Pd) stoichiometry in the Pd(II) complexation process. The single crystal X-ray analysis of Pd(NO_3_)_2_(L-II)_2_ complex confirms a four-coordinated Pd(II) center, with two pyridine nitrogen atoms and two monodentate nitrate oxygens forming a quadrangular geometry. Density functional theory (DFT) calculations further indicate that the formation of 2:1 (L/Pd) complexes is energetically favored, and the stronger basicity of the nitrogen atoms correlates with a higher Pd(II) binding affinity and increased susceptibility to protonation.

## 1. Introduction

Nuclear power, as a high-density and green energy supply with negligible CO_2_ emissions, has long been considered as a promising alternative for traditional fuel energy with the ever-increasing worldwide concern about global warming [1,2,3]. However, the generation of large amounts of nuclear wastes with high radio- and chemo-toxicity, which causes severe threats to biological and environmental systems, has become one of the most pivotal factors stymieing the further development of nuclear industries [4,5,6]. The proper treatment of the harmful fission products (FPs) is an essential yet challenging task for the subsequent disposal of nuclear wastes [7,8,9]. Among these FPs, palladium exists in large quantities up to 1 kg/t in fast reactors at a burn up of 33 GWd/t [10]. These palladium ions can seriously impede the solidification of high-level liquid waste (HLLW), the raffinate of the plutonium and uranium reduction extraction (PUREX) process aiming at removing Pu and U from nuclear wastes, due to the poor solubility of Pd species in the glass matrix [11]. On the other hand, palladium is an important precious metal with widespread applications in the fields of catalysis [12], medical science [13], corrosion resistance alloys [14], electronics [15], etc. However, the extremely low abundance of Pd in the Earth’s crust (less than 3 ng/g) is far from enough to meet the growing industrial demands, leading to an explosive growth in its price in recent years [16]. It is worth noting that only ^107^Pd presents a very weak *β*-emission that is acceptable for most industrial uses, and this isotope only accounts for ca. 17% of the total Pd resources in HLLW [17]. Therefore, the recovery of Pd from HLLW is not only necessary for nuclear waste treatment, but also desirable for commercial interests.

**Figure 1:**
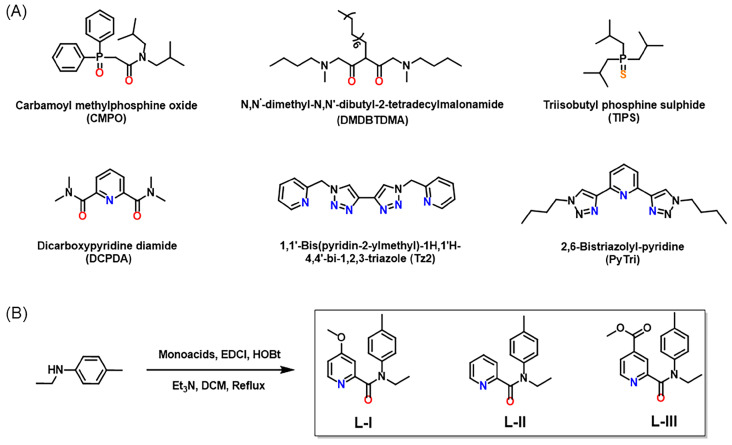
(**A**) Chemical structures of some reported oxygen, sulfur, and nitrogen donor-based ligands; (**B**) synthesis of three nitrogen-based ligands reported in this work.

The solvent extraction technique is currently the best developed method for metal ion separation from HLLW, which possesses attractive merits including easy operation, low energy consumption, and so on [18,19]. This method relies heavily on the use of ligands with high efficiency and selectivity. Different kinds of ligands have been designed for Pd(II) extraction from HLLW in the past years [20] (Figure 1A), mainly focusing on oxygen-based ligands (e.g., CMPO and DMDBTDMA), sulfur-based ligands (e.g., TIPS), and nitrogen-based ligands (e.g., Tz2 [21] and PyTri [22]). However, most oxygen-based ligands inevitably face drawbacks regarding selectivity due to the coextraction towards lanthanides and other fission products, such as Sr(II) and Cs(I) [23,24]. Although sulfur-based ligands exhibit a higher selectivity for Pd(II), they can hardly be employed under highly acidic conditions because of severe protonation [25]. In comparison, nitrogen-based ligands demonstrated a higher potential for Pd(II) separation from simulated HLLW [21,22]. Since Pd(II) belongs to the soft acids, nitrogen donor atoms as soft bases generally exhibit an excellent selectivity towards Pd(II) [26]. In addition, the chemical structures of these nitrogen-based ligands meet the CHON principle, and thus they are more environmentally friendly as no secondary pollution is generated after incineration [27]. It was found that the Pd(II) extraction efficiency of nitrogen-based ligands is largely dependent on their structural characteristics. For example, altering the alkyl side chains can affect the hydrophobicity and steric hindrance of ligands [22]. Diverse types of *n*-heterocycles exhibit different efficiencies for Pd(II) separation [21,28]. Changing the number and direction of nitrogen donors in ligands also results in distinct Pd(II) separation abilities. Disclosing the relationship between ligand structures and Pd(II) complexation and extraction efficiency is an important fundamental task for ligand/material design and the development of related separation processes. However, the influence of the electronic effect of ligands, one of the key factors determining the Pd(II) coordination strength of donor atoms, on palladium complexation and extraction still remains an unexplored territory.

In such a background, this work reports on the design and synthesis of three new pyridine-based ligands with different substituents that show various electronic effects on the nitrogen coordination sites (Figure 1B). **L-I** and **L-III** are decorated with an electron-donating methoxyl group and an electron-withdrawing ester group, respectively, at the *para*-position of the pyridine nitrogen atom, while **L-II** is not substituted. Their Pd(II) extraction ability and complexation mechanisms were investigated comparatively by solvent extraction experiments, spectroscopic methods, single crystal X-ray diffraction analysis, and DFT calculations.

## 2. Results and Discussion

### 2.1. Solvent Extraction

**Figure 2:**
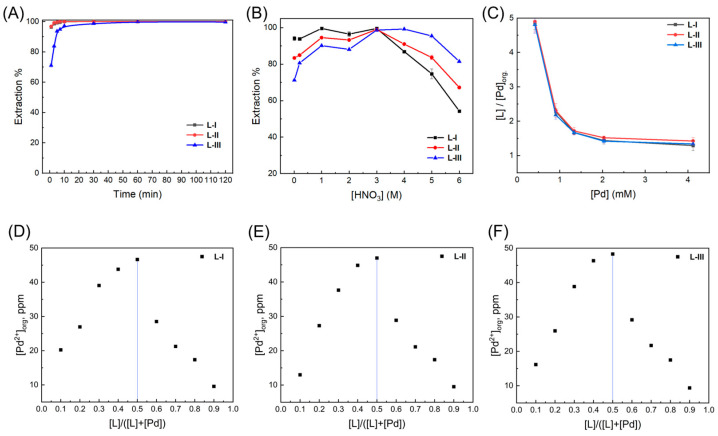
Extraction of Pd(II) by **L-I**, **L-II**, and **L-III**. (**A**) Extraction percentage as function of contact time ([HNO_3_] = 3 M, [**L**] = 2 Mm, [Pd(II)] = 1 mM, 25 °C); (**B**) extraction percentage of Pd(II) as function of HNO_3_ concentration ([**L**] = 2 mM, [Pd(II)] = 1 mM, 25 °C); and (**C**) loading capacities of Pd(II) ([HNO_3_] = 3 M, [**L**] = 2 mM, 25 °C). (**D**–**F**) Job plot analysis ([HNO_3_] = 3 M, [Pd(II)] + [**L**] = 2 mM, 25 °C). Without being specifically stated, organic phase is 3-nitrobenzotrifluoride containing corresponding ligands and aqueous phase is HNO_3_ solution with Pd(NO)_2_.

The solvent extraction behaviors of the ligands toward Pd(II) were investigated starting with extraction kinetics. Given the high solubility of both the extractants and Pd(II)-loaded complexes in 3-nitrobenzotrifluoride, this solvent was selected as the diluent in all extraction experiments to prevent precipitation, emulsification, and third-phase formation. These experiments were carried out by shaking the mixture of the organic phase (solutions of ligands in 3-nitrobenzotrifluoride) and aqueous phase (Pd(NO_3_)_2_ in HNO_3_) with the ratio of 1:1 (*v*/*v*) and detecting the residual Pd(II) concentrations of the aqueous phase at different contact times. As shown in Figure 2A, at 3 M HNO_3_, **L**-**I**, **L**-**II**, and **L**-**III** showed very fast extraction kinetics toward Pd(II) with extraction percentages of over 96% within 1 min. Our previous work suggested that the hydrophobicity of ligands controlled by different alkyl side chains could produce significant differences in Pd(II) extraction kinetics, with the ligand bearing the shortest alkyl side chains showing much faster kinetics [22]. Therefore, one of the determinants responsible for the ultrafast extraction observed here could be the presence of short alkyl side chains in these ligands, which leads to lower hydrophobicity and faster metal ion transfer at the interface during extraction. At equilibrium, all of the three ligands exhibit a quantitative extraction for Pd(II). Based on these results, the extraction time was fixed at 2 h to ensure the equilibria of all the subsequent extraction experiments.

The extraction results at different HNO_3_ concentrations ranging from 0.01 M to 6 M are presented in Figure 2B and Table S4. Despite the presence of similar coordination sites, the three ligands were found to exhibit distinct extraction behaviors toward Pd(II) ions. At 0.01 M HNO_3_, **L**-**I** bearing an electron-donating methoxyl group demonstrates the highest Pd(II) extraction (94%) among the three ligands, while **L-III** with an electron-withdrawing ester group extracts only 71% of the Pd(II). The results suggest that the higher basicity of the nitrogen donor atom produces a positive effect on the Pd(II) extraction due to the stronger binding affinity of donor atoms towards Pd(II). As the aqueous phase is subjected to the change in HNO_3_ concentrations from 0.01 M to 3 M HNO_3_, the three ligands experience an increased extraction for Pd(II) to almost quantitative extraction. This phenomenon can be rationalized by the fact that the increased acidity leads to higher concentrations of NO_3_^−^ anions in the aqueous phase, which facilitates Pd(II) extraction by being co-extracted into the organic phase to keep the charge balance of both aqueous and organic phases [29,30]. When the HNO_3_ concentration is further elevated to 6 M, the Pd(II) extractions by **L-I** and **L-II** decrease dramatically to 54% and 67%, respectively, indicating that the serious protonation of ligands at such a high acidity is the dominating factor to effect the extraction performance. Interestingly, although **L-III** shows a lower extraction efficiency at low acidities (0.01–0.2 M HNO_3_), it exhibits a remarkably higher extraction towards Pd(II) at the acidities ranging from 4 to 6 M than other two ligands. The results above demonstrate a unique but undiscovered phenomenon for Pd(II) extraction systems. At low acidities, the protonation of ligands shows a relatively low impact on Pd(II) extraction. Therefore, the ligands decorated with electron-donating groups exhibit a better extraction ability. However, the pattern is reversed at high acidities, in which cases the protonation of ligands plays the leading role for affecting the extraction, and the donor atoms with higher basicity result in much severer protonation, thus yielding low extraction. This conclusion will be discussed in detail and further verified by DFT calculations (*vide post*). Moreover, we studied the extraction performance and structural changes in three ligands after radiation. After irradiation at 200 kGy, the ^1^H NMR analysis revealed that the structures of the three ligands remained almost unchanged. Additionally, extraction experiments demonstrated that all the three ligands only exhibited a very slight reduction in palladium extraction efficiency (Figures S4–S6 and S10), indicating that these ligands have a good radiation resistance.

**Figure 3:**
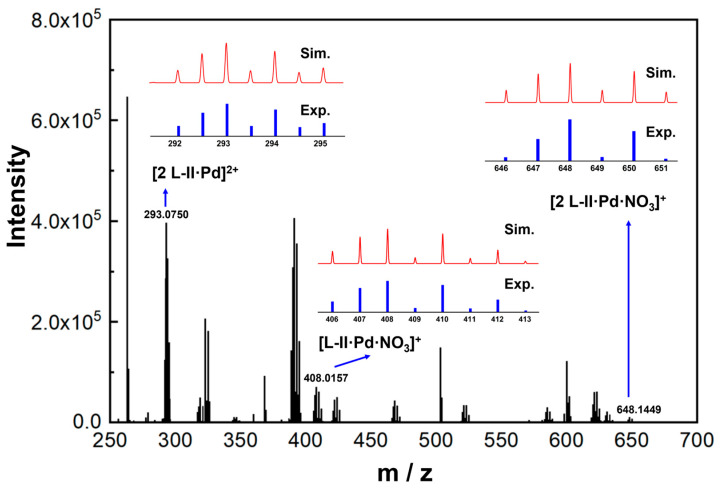
ESI-HRMS spectrum of **L-II**-loaded organic phase(3-nitrobenzotrifluoride) after extraction of Pd(II) ([**L-II**] = 2 mM, [Pd] = 1 mM, and [HNO_3_] = 3 M).

In order to further evaluate the efficiencies of the extraction systems and to probe the stoichiometry of ligands and Pd(II) during extraction, the loading capacities were measured by increasing the concentrations of Pd(II) in the aqueous phase gradually while keeping the concentration of the ligands in organic phases unchanged. As presented in Figure 2C, for the three ligands, when increasing the concentration of Pd(II) from 0.42 mM to 1.33 mM, the [**L**]/[Pd]_org._ values decrease dramatically from ca. 4.9 to 1.1–1.5, reflecting the increased extraction amounts of Pd(II) ions from the aqueous phase to the organic phase. At the Pd(II) concentrations higher than 2.03 mM, the [**L**]/[Pd]_org._ values level off and no significant reduction was further observed, even at 4.12 mM Pd(II). The lowest [**L**]/[Pd]_org._ values for **L-I**, **L-II**, and **L-III** are 1.3, 1.4, and 1.3, respectively, suggesting that each Pd(II) ion could bind to one or two ligands during extraction. These two kinds of stoichiometry, i.e., 1:1 and 2:1 (**L**/Pd), were confirmed by the Job plot method and ESI-HRMS, respectively. As shown in Figure 2D–F, with the change in the [L]/([**L**] + [Pd]) ratio when fixing the total concentration of the ligand and Pd(II) at 2 mM, the amount of extracted Pd(II) into the organic phase changes and exhibits the highest values at [**L**]/([**L**] + [Pd]) = 0.5 for the three ligands, indicating that the main stoichiometry under this condition is 1:1 (**L**/Pd). The ESI-HRMS analysis was performed using **L-II** as the representative ligand (Figure 3). The peaks located at m/z 293.0750, 408.0157, and 648.1449 are assigned to [2 **L-II •** Pd]^2+^, [**L-II** • Pd **•** NO_3_]^+^, and [2 **L-II •** Pd **•** NO_3_]^+^, respectively, confirming the coexistence of the 2:1 and 1:1 (**L**/Pd) complexes during extraction.

**Figure 4:**
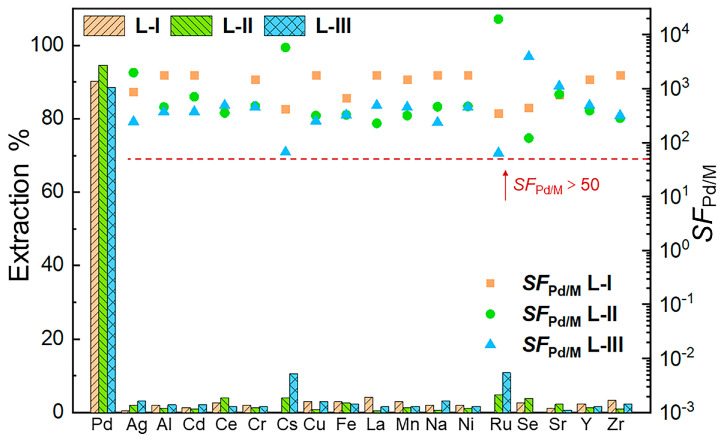
Extraction percentages and *SF*_Pd/M_ values of different ions in simulated HLLW. Organic phase was 2 mM ligand in 3-nitrobenzotrifluoride, and aqueous phase was 100 mg/L metal in 3 M HNO_3_. Forms of all ions used are as follows: Pd^2+^, Ag^+^, Al^3+^, Cd^2+^, Ce^4+^, Cr^3+^, Cs^+^, Cu^2+^, Fe^3+^, La^3+^, Mn^2+^, Na^+^, Ni^2+^, Ru^3+^, SeO_4_^2-^, Sr^2+^, Y^3+^, and ZrO^2+^.

The selectivity of ligands toward Pd(II) was evaluated using a simulated HLLW solution containing 17 competing ions, such as Cs(I), Sr(II), and Ru(III). The composition and concentrations of this solution were similar with those found in actual HLLW (Table S1). Extraction results indicate that the presence of competing ions affects the extraction of Pd(II) to different degrees for the three ligands (Figure 4). **L-I** and **L-II** can recover 90.2% and 94.7% of Pd(II), respectively, with all other competing ions being negligibly extracted (E% < 5%). These results demonstrate an excellent selectivity of **L-I** and **L-II** toward Pd(II). **L-III** also exhibits a good selectivity for Pd(II) over most of the coexisting ions except for a slightly higher extraction for Cs(I) (10.6%) and Ru(II) (10.8%), leading to a relatively lower Pd(II) extraction of 88.6%. As shown in Table S3, the three ligands exhibit markedly higher distribution coefficients for palladium over other metals, confirming their high selectivity for Pd(II). To further investigate the selectivity of these ligands for Pd(II), additional extraction experiments were conducted using Th(IV) and U(VI) as representative radioactive actinides (Figure S11). The results demonstrate that all three ligands exhibited a markedly lower extraction efficiency for these actinides compared to Pd(II), confirming their high selectivity for Pd(II) over actinide species. The separation factors (*SF*_Pd/M_) calculated from the extraction results are all higher than 50, further proving the excellent extraction selectivity of these nitrogen-based ligands for Pd(II). Using **L-II** as the representative ligand, we further performed extraction experiments from simulated HLLW in *n*-octanol, which results in a slightly declined extraction for Pd(II) (83%) and an acceptable selectivity (Figure S12), suggesting that the further replacement of 3-nitrobenzotrifluoride by *n*-octanol is feasible in regard to its lower price.

### 2.2. UV-Vis Spectrophotometric Studies

**Figure 5:**
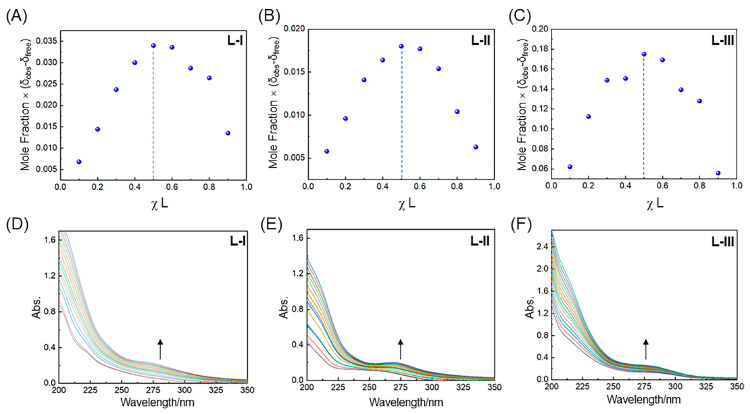
Job plot analysis of stoichiometric ratios between ligands and Pd(II) in acetonitrile at 298 K ([**L**] + [Pd^2+^] = 20 μM): (**A**) **L-I**, (**B**) **L-II**, and (**C**) **L-III**. Stacked UV-Vis titration spectra: (**D**) **L-I**, (**E**) **L-II**, and (**F**) **L-III**.

The complexation behaviors between the ligands and Pd(II) ions in acetonitrile were explored by UV-Vis spectroscopic studies. Job plot experiments were first carried out by fixing the total concentrations of ligand and Pd(II) ions at 20 μM. The results shown in Figure 5A–C indicate the 1:1 complexation stoichiometry for the three ligands with Pd(II). In order to determine the stability constants for these ligands binding to Pd(II), UV-Vis titration experiments were performed in acetonitrile at 20 μM of ligand and varying concentrations of Pd(II) ions from 0 to 30 μM (Figure 5D–F). The stability constants were obtained employing global analysis by the website (http://supramolecular.org/), which follow the order of **L-I** (1.7 × 10^4^) > **L-II** (4.6 × 10^3^) > **L-III** (5.4 × 10^2^) (Figure S13), suggesting again that the high electronegativity of donor atoms is beneficial for complexing metal ions under neutral conditions.

### 2.3. X-Ray Crystallographic Studies

**Figure 6:**
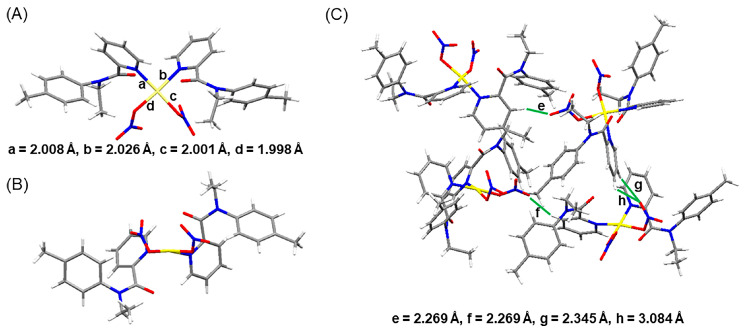
(**A**,**B**) X-ray crystal structure of Pd(NO_3_)_2_(**L-II**)_2_ from different views. (**C**) Packing structure of Pd(NO_3_)_2_(**L-II**)_2_. Gray, white, blue, red, and yellow represent C, H, N, O, and Pd atoms.

In order to achieve more insights into the coordination information between the ligands and Pd(II), a yellowish single crystal of Pd(NO_3_)_2_(**L-II**)_2_ was obtained by slowly evaporating ether into solutions of 2 mM **L-II** and Pd(NO_3_)_2_. The detailed bond information is listed in Table S2. Pd(NO_3_)_2_(**L-II**)_2_ crystallized in the monoclinic *Pca*2*_1_* space group with lattice parameters *a* = 15.9841(5) Å, *b* = 12.9740(5) Å, and *c* = 15.1889(5) Å. In the crystal structure, the central Pd(II) ion is four-coordinated with two nitrogen atoms from pyridine groups and two monodentate nitrate anions in a nearly quadrangular geometry (Figure 6A,B). The *cis* configuration of the complex is likely favored due to reduced steric hindrance compared with the *trans* isomer. On account of the presence of nitrate anions, the Pd(NO_3_)_2_(**L-II**)_2_ complex can further stack through intermolecular hydrogen bonds between nitrate oxygen atoms and pyridine hydrogen atoms (Figure 6C).

### 2.4. Structures Simulations and Binding Energies Calculations

**Figure 7:**
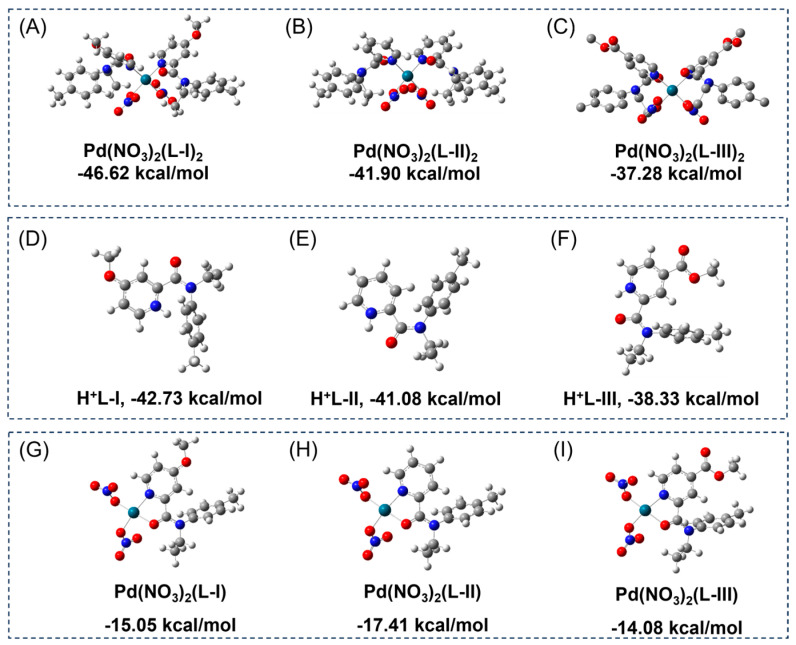
DFT optimized structures and binding energies of (**A**) Pd(NO_3_)_2_(**L-I**)_2_, (**B**) Pd(NO_3_)_2_(**L-II**)_2_, and (**C**) Pd(NO_3_)_2_(**L-III**)_2_. Optimized protonated structures of ligands and protonation energies of (**D**) **L-I**, (**E**) **L-II**, and (**F**) **L-III**. Optimized structures and binding energies of (**G**) Pd(NO_3_)_2_(**L-I**), (**H**) Pd(NO_3_)_2_(**L-II**), and (**I**) Pd(NO_3_)_2_(**L-III**).

The different electronegativity of the pyridine nitrogen atoms of the three ligands was confirmed by DFT calculations. As shown in Figure S14, the natural charges on pyridine nitrogen atoms obtained from the optimized structures follow the order of **L-III** (−0.441) > **L-II** (−0.446) > **L-I** (−0.472), suggesting the successful control of the nitrogen basicity by introducing electro-donating or electro-withdrawing groups. The binding energies of Pd(NO_3_)_2_**L**_2_ complexes in acetonitrile were firstly compared according to DFT calculations. The structural optimization was carried out at the B3LYP/6-31G* level of the theory referring to the geometry of single crystals. As shown in Figure 7A–C, the binding energies of Pd(NO_3_)_2_(**L-I**)_2_, Pd(NO_3_)_2_(**L-II**)_2_, and Pd(NO_3_)_2_(**L-III**)_2_ are calculated to be −46.62 kcal/mol, −41.90 kcal/mol, and −37.28 kcal/mol, respectively, suggesting again that the increased electronegativity is beneficial for Pd(II) complexation in neutral conditions. These results can also rationalize the trend observed from solvent extraction experiments at low acidities. However, the extraction abilities of the ligands at high acidities (>3 M HNO_3_) follow the trend of **L-III** > **L-II** > **L-I**, which is the opposite of that at low acidities. This could be owing to the higher degree of protonation of the donor atoms with higher electronegativity. To prove this speculation, the protonation energies of the three ligands were calculated. As shown in Figure 7D,E, the protonation energy of **L-I** (−42.73 kcal/mol) is a little bit higher than that of **L-II** (−41.08 kcal/mol), indicating that the higher electronegativity leads to easier protonation. Compared with **L-I** and **L-II**, **L-III** has a higher protonation energy (−38.33 kcal/mol), which suggests that **L-III** is much more difficult to be protonated and this is in great agreement with its markedly higher extraction ability for Pd(II) at high acidities.

Since the formation of 1:1 (**L**/Pd) complexes was demonstrated in Job plot experiments, their possible configurations were also optimized referring to reported structures of picolinamide with metal ions [31]. As presented in Figure 7G–I, these ligands may also bind to Pd(II) ions according to the pyridine nitrogen and amide oxygen in a bidentate manner, with the cooperation of two monodentate nitrate anions. However, their binding energies (−15.05 kcal/mol for Pd(NO_3_)_2_(**L-I**), −17.41 kcal/mol for Pd(NO_3_)_2_(**L-II**), and −14.08 kcal/mol for Pd(NO_3_)_2_(**L-III**)) are profoundly higher than those of Pd**L**_2_ complexes, indicating that the formation of Pd**L**_2_ complexes is more energetically favored. Furthermore, the double free energy difference (ΔΔ*E*) values between Pd(NO_3_)_2_(L)_2_ and protonated **L** are −3.89, −0.82, and 1.05 kcal/mol for ligands **L-I**, **L-II**, and **L-III**, respectively. These results suggest that **L-I** and **L-II** exhibit a greater propensity to form 2:1 complexes with Pd(II) compared with **L-III** [32].

## 3. Materials and Methods

### 3.1. General

Chemical reagents used for ligand synthesis were purchased from Energy Chemical Co., Ltd. (analytically pure, Shanghai, China) and Sigma-Aldrich (Shanghai, China) in their purest forms. Metal nitrates in analytical purity utilized in extraction experiments were provided by Aladdin Industrial Corporation (Chengdu, China). Organic solvents employed in spectroscopic experiments were purchased from Chengdu Chron Chemicals Co., Ltd., Chengdu, China.

NMR experiments were carried out with a Bruker AVANCE AV II-400 MHz spectrometer (Bruker, Karlsruhe, Germany). UV-Vis data were collected on a SHIMADZU UV-2600i (Kyoto, Japan). The concentrations of metal ions were examined by a PerkinElmer ICP optima 8000 (Waltham, MA, USA). ESI-HRMS data were collected on a Bruker Daltonics Micro TOF-Q II (SHIMADZU, Kyoto, Japan).

### 3.2. Synthesis of Ligands

The ligands were synthesized through condensation reactions between *N*-ethyl-4-methylaniline and different monoacid precursors. In a general procedure, *N*-ethyl-4-methylaniline (1.00 g, 7.40 mmol), monoacids (7.40 mmol), EDCI (1.70 g, 8.88 mmol), HOBt (1.09 g, 8.88 mmol), and Et_3_N (0.899 g, 8.88 mmol) were mixed in 50 mL of DCM. The solution was refluxed for 24 h at 45 °C under Ar atmosphere and then was washed with water (50 mL × 3). The pure products were collected by fast column chromatography (DCM/MeOH, 30:1, *v*/*v*), and the detailed characterization data and figures are presented in Supplementary Materials.

### 3.3. Extraction Procedure

In solvent extraction experiments, the organic phase was prepared by dissolving extractants into 3-nitrobenzotrifluoride, which was selected as the diluent in this work due to its perfect solubility towards all these ligands and their extracted species. Before extraction, the organic phase was contacted with the HNO_3_ solution of corresponding concentrations with the ratio of 1:1 (*v*/*v*). The aqueous phase containing 1 mM of Pd(II) was prepared by dissolving Pd(NO_3_)_2_ in nitric acid (1–6 M). In a general extraction procedure, the organic and aqueous phases were mixed (0.5 mL/0.5 mL) and shaken vigorously for a required period of time. Then, the concentrations of Pd(II) ions in aqueous phases were tested by ICP-AES or ICP-MS. The values of extraction percentages (*E*%), distribution ratios (*D*), and *SF*_Pd/M_ of Pd(II) were obtained according to the Equations (1), (2), and (3), respectively:

(1) $$E\;\%=\frac{c_{org.}}{c_{tot.}}$$

(2) $$D=\frac{c_{org.}}{c_{aq.}}$$

(3) $${SF}_{Pd/M}=\frac{D_{Pd}}{D_{M}}$$

where *c_aq._*, *c_org._*, and *c_tot._* stand for the concentration of Pd(II) ions in the aqueous phase, organic phase, and the initial Pd(II) concentrations in aqueous phase before extraction, respectively.

### 3.4. DFT Calculations

DFT calculations were conducted employing Gaussian 09 package [33]. Becke-Lee-Young-Parr [34,35] (B3LYP)/SDD-6-31G* was used for structural optimization in the gas phase, in which the 6-311++G** basis set was used for the C, H, O, and N, and the SDD basis set was used for Pd(II). Calculations were performed without any symmetry restrictions. The minimum energies of the optimized structures were confirmed by frequency calculations. The single-point energies were calculated in acetonitrile using the SMD solvation model at the B3LYP/SDD-6-311++G** level of theory. The binding energies were calculated as the differences in total energies of products and reactants. For the energy calculations of generated Pd^2+^ complexes, Pd(NO_3_)_2_(H_2_O)_2_ was used as the raw reagent as it is the main species in HNO_3_ solution (Figure S15). For the calculation of protonation energies, H_3_O^+^ was used as the raw reagent.

## 4. Conclusions

Three new nitrogen-based ligands were designed and synthesized for the efficient separation of palladium from HLLW. The electronegativity of nitrogen donor atoms was manipulated by introducing substituents with different electronic effects. The results from solvent extraction experiments indicate that anchoring an electron-donating group to the pyridyl group benefits the extraction of Pd(II) at low acidities but decreases its performance under highly acidic conditions, while reducing the electronegativity of the nitrogen donor by adding an electron-withdrawing group at the *para* position results in a poorer extraction at low acidities but much better performance at high acidities. These unique results implicate that the coordination strength between the donor atoms and metal ions dominates the extraction efficiency at low acidity, while the protonation of ligands becomes the main factor affecting the extraction performance at high acidity. Both 1:1 and 2:1 (**L**/Pd) complexes can be formed for each of the ligands as indicated by the Job plot method and ESI-HRMS. The single crystal structure of the Pd(NO_3_)_2_(**L-II**)_2_ complex reveals a four-coordination mode of Pd(II) with two ligands cooperatively coordinated with two monodentate nitrate anions in a quadrilateral fashion. DFT calculations demonstrate the preferential formation of 2:1 (**L**/Pd) complexes, and the stronger basicity of nitrogen atoms leads to a higher binding affinity but easier protonation, which are congruent with the results from solvent extraction experiments. This work provides several highly efficient ligands for palladium separation from HLLW, and more importantly, demonstrates the significant influence of the electronic effect on Pd(II) complexation and extraction.

## Data Availability

Data are contained within the article and Supplementary Materials.

## Supplementary Materials

The following supporting information can be downloaded at https://www.mdpi.com/article/10.3390/molecules30071533/s1: Figure S1: ^1^H NMR spectrum of **L-I**, Figure S2: ^1^H NMR spectrum of **L-II**, Figure S3: ^1^H NMR spectrum of **L-III**, Figure S4: ^1^H NMR spectrum of **L-I** after irradiation, Figure S5: ^1^H NMR spectrum of **L-II** after irradiation, Figure S6: ^1^H NMR spectrum of **L-III** after irradiation, Figure S7: ^13^C NMR spectrum of **L-I**, Figure S8: ^13^C NMR spectrum of **L-II**, Figure S9: ^13^C NMR spectrum of **L-III**, Figure S10: Pd(II) extraction percentage before and after irradiation, Figure S11: Extraction of Pd^2+^, Th^4+^, and UO_2_^2+^, Figure S12: Extraction of different metal ions in simulated HLLW with **L-II**, Figure S13: Determination of the stability constants for (A) **L-I**; (B) **L-II**; (C) **L-III**, Figure S14: Structural optimization and natural charges on pyridine nitrogen atoms, Figure S15: Optimized geometries of (A) H_2_O, (B) H_3_O^+^, (C) NO_3_^−^, and (D) Pd(NO_3_)_2_(H_2_O)_2_ for binding energy calculations, Table S1: Concentrations of the metal elements in simulated HLLW (3 M HNO_3_), Table S2: Crystal data and structure refinements for Pd(NO_3_)_2_(**L-II**)_2_, Table S3: The distribution ratio to different ions with three ligands in simulated HLLW, and Table S4: The distribution ratio of Pd(II) with three ligands at different HNO_3_ acidities.
