# Supplementary material for: Manipulating Electronic Effect of Nitrogen Donor-Based Ligands for Efficient Complexation and Separation of Palladium from Highly Acidic Solution
Source: Molecules. 2025 Mar 30;30(7):1533. doi: 10.3390/molecules30071533 (PMC11990395; doi:10.3390/molecules30071533)
Supplement: Supplementary file 1 [file molecules-30-01533-s001.zip › molecules-3504714-supplementary.pdf]

Supporting Information

Manipulating Electronic Effect of Nitrogen Donor-Based Ligands for Efficient Complexation and Separation of Palladium from Highly Acidic Solution

Yuyang Gan †, Yimin Cai †, Song Huang, Xiaowei Li *, Wen Feng and Lihua Yuan*

College of Chemistry, Key Laboratory of Radiation Physics and Technology of the Ministry of Education, Institute of Nuclear Science and Technology, Sichuan University, Chengdu 610064, China; 19981458619@163.com (Y.G.); ymcai@scu.edu.cn (Y.C.); anonunknown@163.com (S.H.); wfeng9510@scu.edu.cn (W.F.)

* Correspondence: lixw@scu.edu.cn (X.L.); lhyuan@scu.edu.cn (L.Y.)

† These authors contributed equally to this work.

Contents

1. Ligand characterization data…………………………………….……………….Page 3
2. ^{1}H NMR spectra ……………….…………………………………….…………..Page 4
3. ^{13}C NMR spectra ……………….……………………………………………….Page 6
4. Irradiation experiments………………………...Page 7
5. Selectivity test…………………………………………………………………....Page 7
6. Extraction of different metal ions in simulated HLLW with **L-II** in *n*-octanol…….Page 8
7. Determination of the stability constants……….. …………………………………Page 8
8. Optimized geometries for binding energy calculations…………………………..….Page 8
9. Structural optimization and natural charges on pyridine nitrogen atoms………..….Page 9
10. Concentrations of the metal elements in simulated HLLW…………………………Page 9
11. The distribution ratio …………………………………..………………………....Page 11

Ligand characterization.

L-I: White powder (1.51 g, 76%). ^{1}H NMR (400 MHz, 298 K, $CDCl_3$): δ 8.13 (d, *J* = 8.0 Hz, 1H), 6.90-7.00 (m, 5H), 6.60 (d, *J* = 8.0 Hz, 1H), 3.96 (q, *J* = 7.2 Hz, 2H), 3.73 (s, 3H), 2.25 (s, 3H), 1.20-1.23 (t, *J* = 7.2 Hz, 3H). ^{13}C NMR (100 MHz, 298 K, $CDCl_3$): δ 168.3, 165.6, 156.3, 149.7, 139.9, 136.5, 129.5, 127.5, 110.2, 109.3, 55.2, 44.9, 20.9, 12.7. ESI-MS calc. for $C_{16}H_{18}N_2O_2$ [**L-I** + H]$^+$: *m/z* = 271.1368, found 271.1417.

L-II: White powder (1.34 g, 75%). ^{1}H NMR (400 MHz, 298 K, $CDCl_3$): δ 8.41 (m, 1H), 7.53 (m, *J* = 8.0 Hz, 1H), 7.33 (m, *J* = 8.0 Hz, 1H), 7.11 (m, 1H), 6.93-6.97 (q, J_1 = 24.0 Hz, J_2 = 8.0 Hz, 4H), 4.01 (q, *J* = 8.0 Hz, 2H), 2.25 (s, 3H), 1.23 (t, *J* = 7.2 Hz, 3H). ^{13}C NMR (100 MHz, 298 K, $CDCl_3$): δ 168.4, 154.8, 148.6, 139.9, 136.5, 136.0, 129.6, 127.6, 123.6, 123.4, 44.9, 20.9, 12.8. ESI-MS calc. for $C_{15}H_{16}N_2O$ [**L-II** + H]$^+$: *m/z* = 241.1263, found 241.1328.

L-III: White powder (1.45 g, 81%). ^{1}H NMR (400 MHz, 298 K, $CDCl_3$): δ 8.37-8.38 (d, *J* = 4.0 Hz, 1H), 7.84 (s, 1H), 7.54-7.55 (d, *J* = 4.0 Hz, 1H), 6.83-6.89 (q, *J* = 8.0 Hz, 4H), 3.86-3.92 (m, 2H), 3.81 (s, 3H), 2.13 (s, 3H), 1.13-1.16 (m, 3H). ^{13}C NMR (100 MHz, 298 K, $CDCl_3$): δ 168.5, 166.1, 158.5, 149.3, 139.8, 136.1, 131.3, 127.7, 121.3, 53.2, 44.4, 30.1, 21.3, 13.1. ESI-MS calc. for $C_{17}H_{18}N_2O_3$ [**L-III** + H]$^+$: *m/z* = 299.1390, found 299.1379.

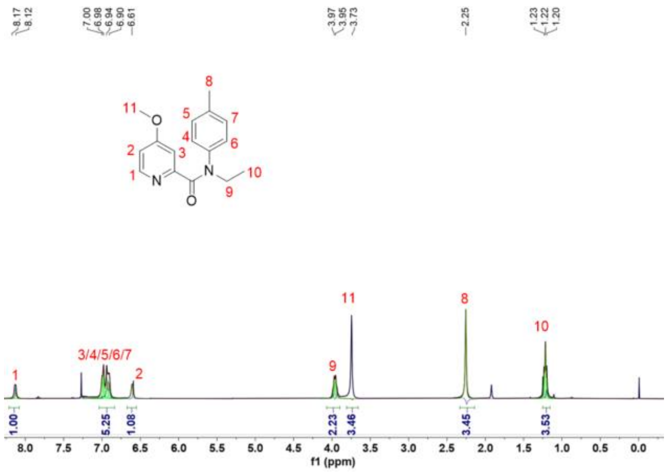

Figure S1 ^{1}H NMR spectrum of **L-I** in $CDCl_3$ (400 MHz, 298 K).

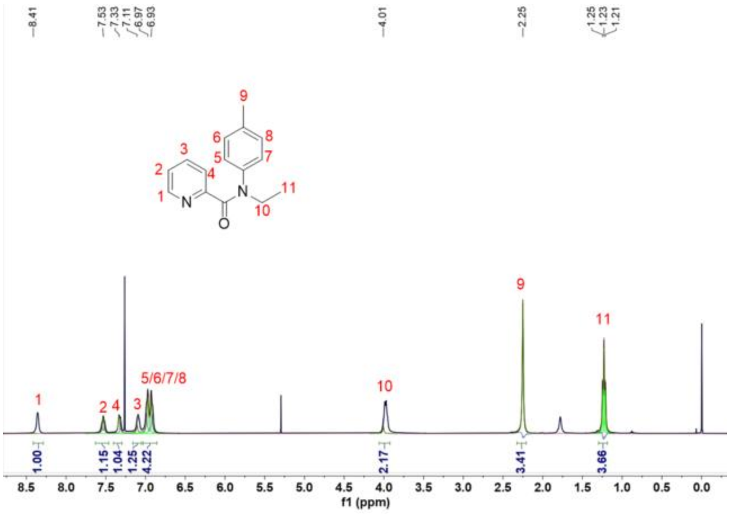

Figure S2 ^{1}H NMR spectrum of **L-II** in $CDCl_3$ (400 MHz, 298 K).

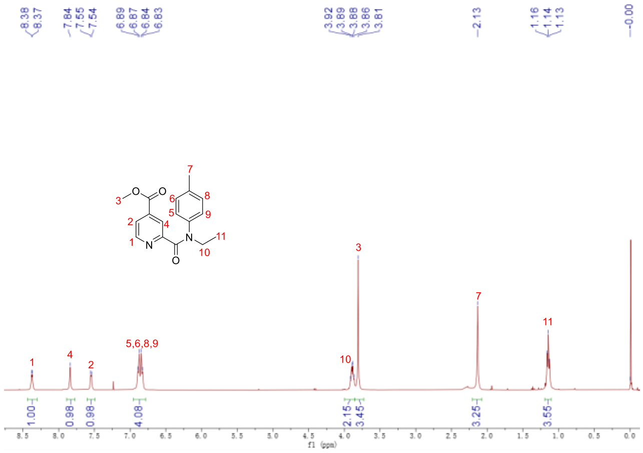

Figure S3 ^{1}H NMR spectrum of **L-III** in $CDCl_3$ (400 MHz, 298 K).

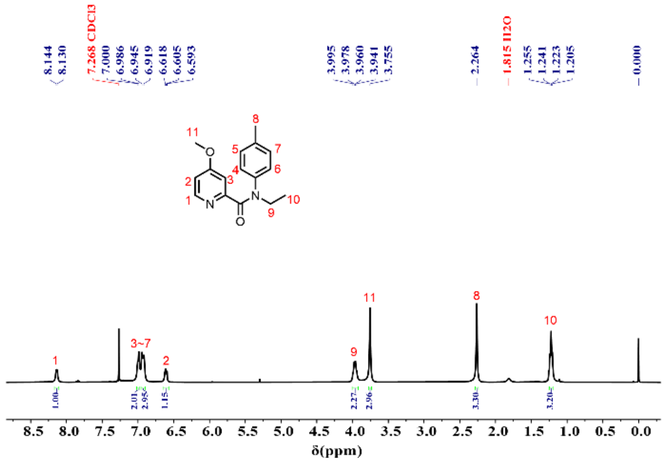

Figure S4 ^{1}H NMR spectrum of **L-I** in $CDCl_3$ (400 MHz, 298 K) after irradiation.

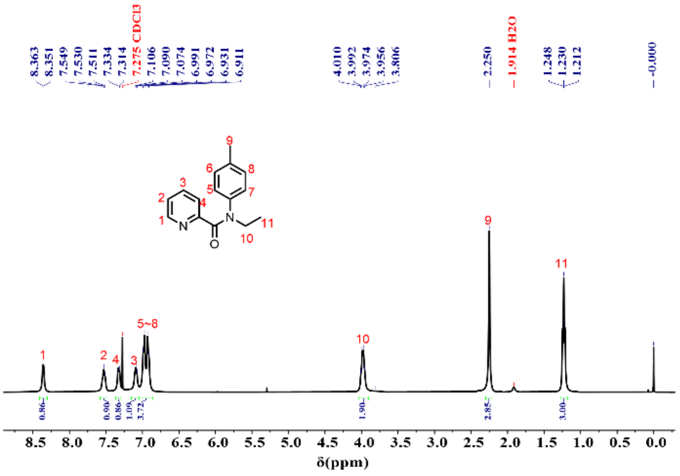

Figure S5 ^{1}H NMR spectrum of **L-II** in $CDCl_3$ (400 MHz, 298 K) after irradiation.

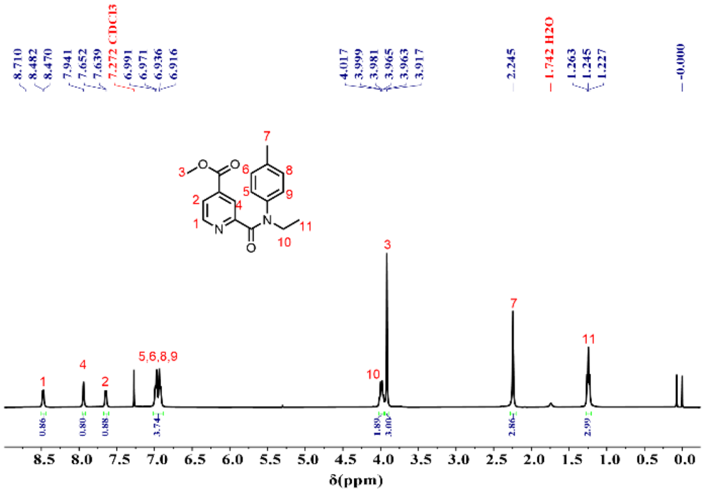

Figure S6 ^{1}H NMR spectrum of **L-III** in $CDCl_3$ (400 MHz, 298 K) after irradiation.

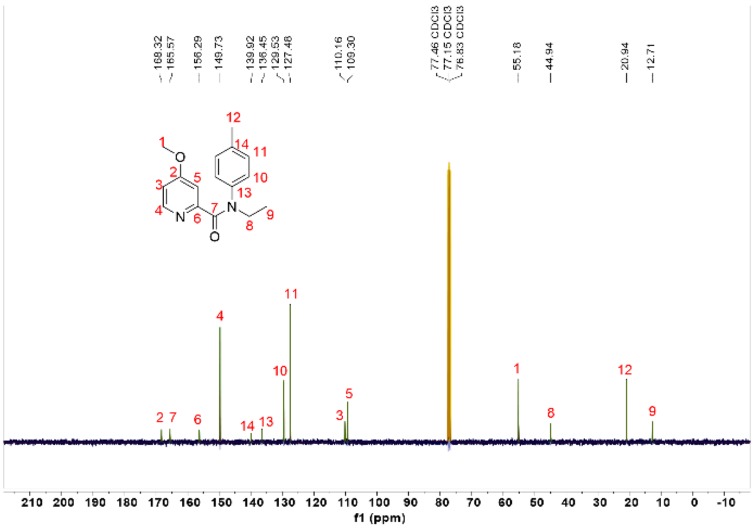

Figure S7 ^{13}C NMR spectrum of **L-I** in $CDCl_3$ (100 MHz, 298 K).

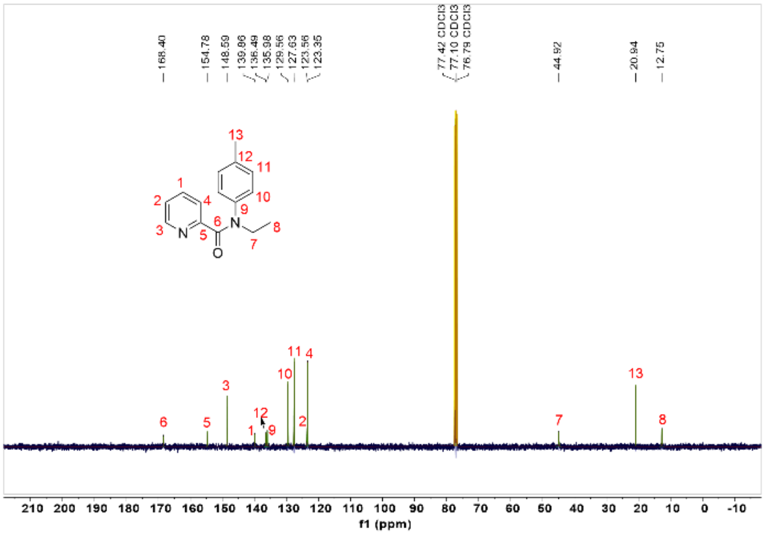

Figure S8 ^{13}C NMR spectrum of **L-II** in $CDCl_3$ (100 MHz, 298 K).

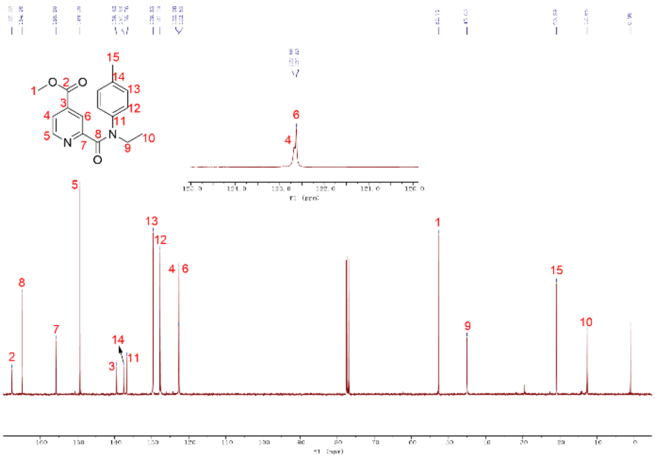

Figure S9 ^{13}C NMR spectrum of **L-III** in $CDCl_3$ (100 MHz, 298 K).

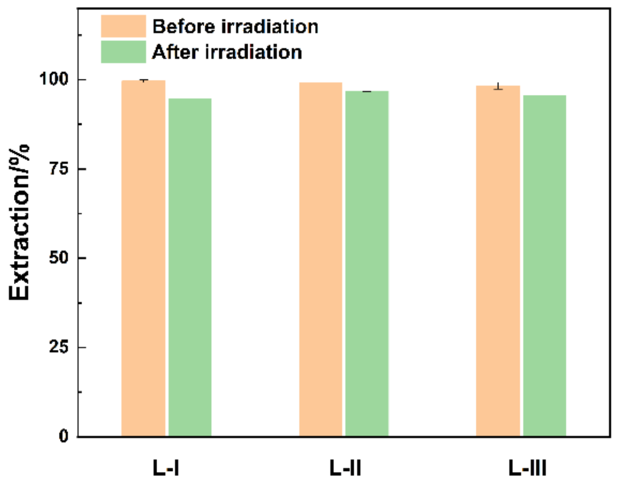

Figure S10. Pd(II) extraction percentage of three ligands before and after irradiation at 25 °C ([**L**] = 2 mM in 3-nitrobenzotrifluoride, [Pd(II)] = 1 mM, [HNO_3] = 3 M).

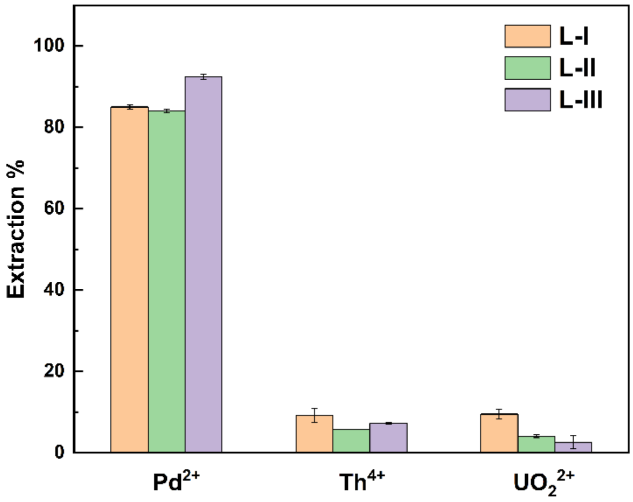

Figure S11. Extraction of Pd^{2+}, Th^{4+}, and UO_2^{2+} with three ligands ([**L**] = 2 mM in 3-nitrobenzotrifluoride, [HNO_3] = 3 M).

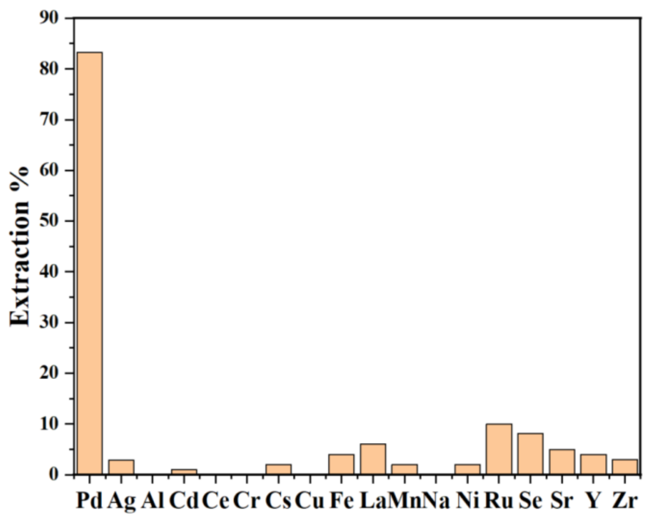

Figure S12. Extraction of different metal ions in simulated HLLW with **L-II** in *n*-octanol ([**L-II**] = 2 mM, 3 M HNO_3).

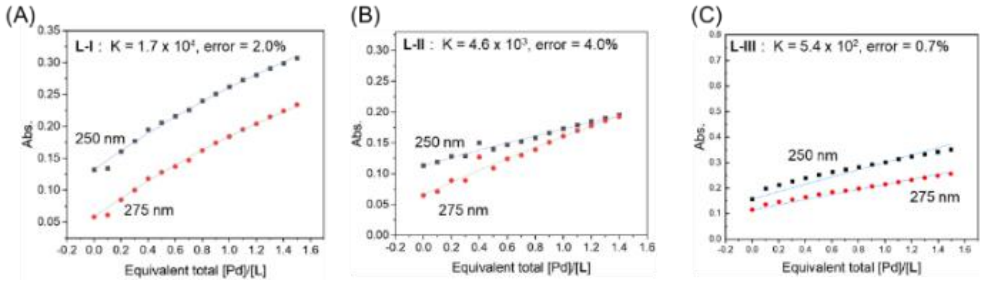

Figure S13. Determination of the stability constants in CH_3CN at 298 K ([**L**] = 20 μM). (A) **L-I**; (B) **L-II**; (C) **L-III**.

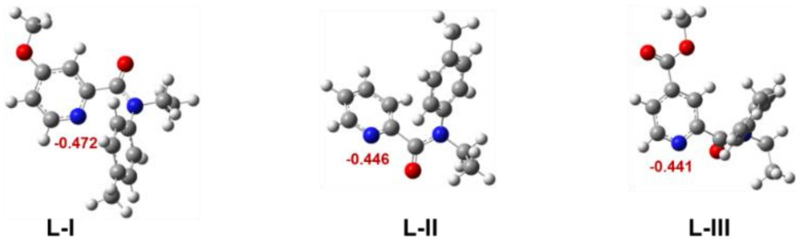

Figure S14. Structural optimization and natural charges on pyridine nitrogen atoms.

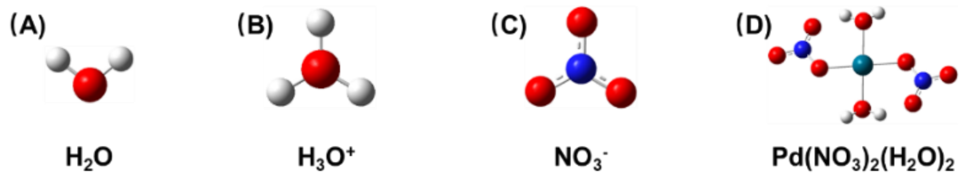

Figure S15. Optimized geometries for binding energy calculations. (A) H_2O, (B) H_3O^+, (C) NO_3^-, and (D) $Pd(NO_3)_2(H_2O)_2$.

Table S1. Concentrations of the metal elements in simulated HLLW (3 M HNO_3).

Metal ion	Concentration (ppm)	Metal ion	Concentration (ppm)
Pd (II)	90.0	Mn (II)	363.6
Ag (I)	6.2	Na (I)	151.4
Al (III)	17.2	Sr (II)	27.4
Ce (IV)	237.2	Y (III)	47.0
Cr (III)	21.0	Zr (IV)	38.4
Fe (III)	444.4	Cs (I)	288.6
La (III)	97.3	Cd (II)	6.0
Cu(II)	5.0	Ni(II)	9.7
Ru(III)	172.4	Se(IV)	6.6

Table S2. Crystal data and structure refinements for $Pd(NO_3)_2$(**L-II**)$_2$.

	$Pd(NO_3)_2$(**L-II**)$_2$
CCDC	2340387
Empirical formula	$C_{30}H_{32}N_6O_8Pd$
Formula weight	711
Temperature/K	100
Crystal system	orthorhombic
Space group	*Pca2₁*
a/Å	15.9841(5)
b/Å	12.9740(5)
c/Å	15.1889(5)
α/°	90
β/°	90
γ/°	90
Volume/Å^3	3149.84(19)
Z	4
ρ_{calc}g/cm^3	1.499
μ/mm^{-1}	0.648
F(000)	1456.0
Crystal size/mm^3	0.15 × 0.1 × 0.1
Radiation	MoKα (λ = 0.71073)
2θ range for data collection/°	3.14 to 58.68
Index ranges	–20 ≤ h ≤ 19, –15 ≤ k ≤ 13, –20 ≤ l ≤ 14
Reflections collected	18335
Independent	6022 [R_{int} = 0.0358,

reflections	R_{sigma} = 0.0414]
Data/restraints/parameters	6022/653/484
GOF on F^2	1.026
Final R indexes [I>=2σ (I)]	R_1 = 0.0493, wR_2 = 0.1100
Final R indexes [all data]	R_1 = 0.0696, wR_2 = 0.1243
Largest diff. peak/hole / e Å^{-3}	1.17/–1.32

Table S3. The distribution ratio to different ions with three ligands in simulated HLLW ([L] = 2 mM in 3-nitrobenzotrifluoride, [HNO_3] = 3 M).

**Ligands** / **Metal ions**	**L-I**	**L-II**	**L-III**
Pd (II)	9.2212	17.7439	7.787346
Ag (I)	0.0047	0.020408	0.031992
Al (III)	0.0211	0.012002	0.02145
Cd (II)	0.0135	0.010101	0.02145
Ce (IV)	0.0276	0.041667	0.01626
Cr (III)	0.0201	0.012428	0.017294
Cs (I)	0.0016	0.041667	0.118568
Cu (II)	0.0311	0.00853	0.030928
Fe (III)	0.0300	0.02646	0.023541

La (III)	0.0443	0.005025	0.01626
Mn (II)	0.0310	0.012915	0.017294
Na (I)	0.0209	0.006512	0.033058
Ni (II)	0.0201	0.011366	0.017294
Ru (III)	0.0005	0.051245	0.121076
Se (IV)	0.0272	0.039501	0.002004
Sr (II)	0.0120	0.023239	0.007049
Y (III)	0.0242	0.012288	0.01626
Zr (IV)	0.0340	0.009855	0.02459

Table S4. The distribution ratio of Pd(II) with three ligands at different HNO_3 acidities ([**L**] = 2 mM, [Pd(II)] = 1 mM, 25 °C).

Ligands / Acidity/M	L-I	L-II	L-III
0.2	15.28234896	5.644346	4.165289
1	> 99	17.35075	9.162602
2	27.60191004	13.82136	7.417508
3	> 99	> 99	76.51938
4	6.639471929	10.09976	> 99
5	2.96039604	5.121333	21.04284
6	1.187684039	2.051423	4.412773
